# Microneedle Patches with Antimicrobial and Immunomodulating Properties for Infected Wound Healing

**DOI:** 10.1002/advs.202300576

**Published:** 2023-05-18

**Authors:** Shengbo Li, Xuemei Wang, Zhiyao Yan, Tian Wang, Zhenbing Chen, Heng Song, Yongbin Zheng

**Affiliations:** ^1^ Department of Gastrointestinal Surgery, Renmin Hospital of Wuhan University, College of Chemistry & Molecular Science, Institute of Molecular Medicine Wuhan University Wuhan 430060 China; ^2^ Medical College Hubei University of Arts and Science Xiangyang 441000 China; ^3^ Department of Hand Surgery, Union Hospital, Tongji Medical College Huazhong University of Science and Technology Wuhan 430022 China

**Keywords:** antimicrobial, dopamine, immunity, infected wound healing, microneedle

## Abstract

Treatment of infected wounds remains a challenge owing to antibiotic resistance; thus, developing smart biomaterials for the healing of infected wounds is urgently needed. In this study, a microneedle (MN) patch system with antimicrobial and immunomodulatory properties is developed to promote and accelerate infected wound healing. In the MN patch (termed PFG/M MNs), a nanoparticle with polydopamine (PDA)‐loaded iron oxide is grafted with glucose oxidase (GOx) and hyaluronic acid (HA) and then integrated into the tips, and amine‐modified mesoporous silica nanoparticles (AP‐MSNs) are incorporated into the bases. Results show that PFG/M MNs eradicate bacterial infections and modulate the immune microenvironment, combining the advantages of chemodynamic therapy, photothermal therapy, and M2 macrophage polarization from Fe/PDA@GOx@HA in the tips as well as anti‐inflammatory effect of AP‐MSNs from the MN bases. Thus, the PFG/M MN system is a promising clinical candidate for promoting infected wound healing.

## Graphical abstract

A novel microneedle system with excellent antimicrobial and immunomodulatory properties is designed and developed. The microneedle system penetrates the bacterial biofilm to efficiently deliver nanomaterials. Under NIR laser irradiation, this system can be integrated into multiple treatment methods (chemodynamic therapy, photothermal therapy, and immunomodulation).

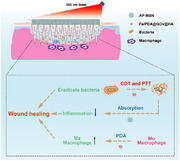

## 1 Introduction

The integrity and protective function of the human skin are often compromised by burns, trauma, surgery, and chronic diseases such as diabetes. Skin wounds are susceptible to various bacterial pathogens, leading to wound infections. Wound infection prolongs healing time, seriously affects the quality of life of patients, and significantly increases the economic burden and risk of mortality.^[^ ^1^ ^]^ It is becoming more challenging as antibiotic resistance complicates the treatment of infected wounds. Thus, developing novel therapies for treating infected wounds is urgently needed.

Bacterial biofilm formation is an important cause of persistent infection, significantly inhibiting wound healing. The composition and organization of biofilms confer a natural barrier to the diffusion of antibacterial agents, leading to antibiotic tolerance and resistance.^[^ ^2^ ^]^ The microenvironment of an infected wound features a low pH, hypoxia, and high levels of glutathione (GSH) and H_2_O_2_.^[^ ^3^ ^]^ Microenvironment‐activated antimicrobial agents have great therapeutic potential for the treatment of infected wounds.^[^ ^4^ ^]^ Strategies for pathological microenvironment remodeling, including photothermal and photodynamic antimicrobial therapy, local oxygen delivery, and reactive oxygen species scavenging, promote chronic wound healing.^[^ ^5^ ^]^

Some nanomaterials such as metal oxide nanoparticles (NPs) exhibit unique, pathologically switchable, and multienzyme‐like properties. Nanozymes effectively promote infected wound healing through chemodynamic therapy (CDT).^[^ ^3a^ ^]^ Photothermal therapy (PTT) is a minimally invasive technique based on photochemical reactions that prevent drug resistance by converting light energy (usually near‐infrared light) into heat energy, causing bacterial death. The combination of CDT with PTT can result in synergistic antimicrobial effects.^[^ ^6^ ^]^ In addition, excessive M1 macrophage activation and pro‐inflammatory factor release cause chronic non‐healing wound. Microenvironment immunomodulation is also an effective strategy for healing chronic wounds.^[^ ^7^ ^]^ An integrated system that combines immunomodulation, PTT, and CDT is expected to act on multiple targets simultaneously, thereby greatly enhancing the synergistic effect of promoting wound healing. However, such system is still lacking.

A microneedle (MN), which consists of a needle array and a base, is a minimally invasive and effective transdermal drug delivery system for promoting wound healing.^[^ ^8^ ^]^ Needle tips can penetrate and disrupt the integrity of biofilms, contributing to the diffusion of antibacterial agents into the infected wounds,^[^ ^9^ ^]^ which will greatly improve the antimicrobial effect. However, degraded bacteria and necrotic cells release pathogenic nucleic acid fragments in infected wounds in this antimicrobial process, eliciting potent inflammatory responses that lead to excessive production of inflammatory cytokines and prolonged immune activation.^[^ ^10^ ^]^ Chronic inflammation is a significant contributor to non‐healing wounds.^[^ ^11^ ^]^ Until now, most biomaterials with antibacterial properties rarely have the function of removing pro‐inflammatory factors such as free nucleic acids. Therefore, the MN patch holds a promise for practical applications that can effectively act as an antibacterial and adsorb pro‐inflammatory factors, such as free nucleic acids, for inflammatory responses.

**Scheme 1:**
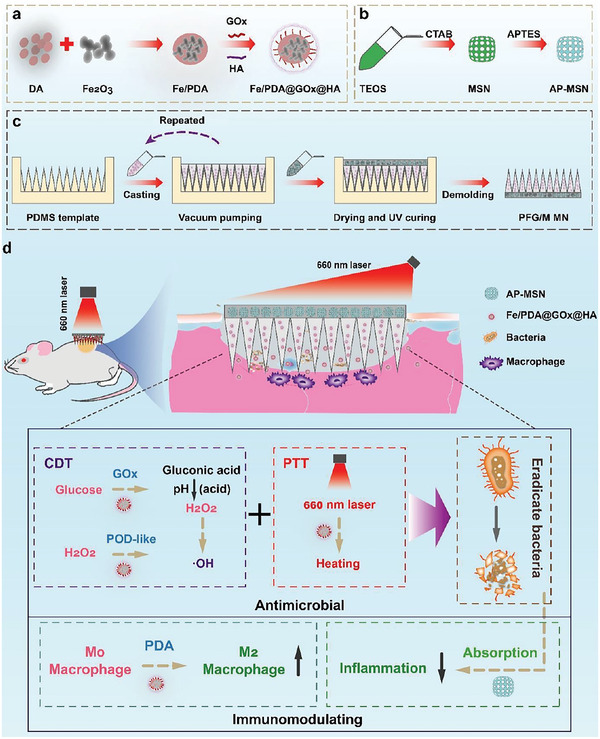
Schematic illustration of the preparation of a) Fe/PDA@GOx@HA, b) AP‐MSN, c) PFG/M MN, and d) therapeutic mechanism of PFG/M MN for infected wound healing. TEOS: tetraethyl orthosilicate; CTAB: cetyltrimethylammonium bromide; APTES: 3‐aminopropyltriethoxysilane.

In this study, we aimed to design portable MNs exhibiting antibacterial and immunomodulatory properties in infected wounds. We encapsulated novel NPs (Fe/PDA@GOx@HA) and amine‐modified mesoporous silica NPs (MSNs) in the tips and bases of the MNs, respectively (termed PFG/M MNs, **Scheme** **1**). We successfully prepared Fe/PDA@GOx@HA with polydopamine (PDA)‐loaded iron oxide as the core, which was transplanted with glucose oxidase (GOx) and hyaluronic acid (HA). The MN tips penetrated the biofilms, resulting in the diffusion of Fe/PDA@GOx@HA into the infected wounds. Fe/PDA@GOx@HA decomposed under the high GSH and weak acid conditions of the infected wound microenvironment, thereby releasing GOx, iron ions, and PDA. GOx converts glucose to gluconic acid and produces hydrogen peroxide, which acts as a substrate for Fe‐catalyzed CDT.^[^ ^12^ ^]^ In addition, the released PDA exhibited excellent photothermal properties under laser irradiation, thereby achieving a synergistic antimicrobial effect by combining CDT and PTT and promoting M2 macrophage polarization to enhance wound repair.^[^ ^13^ ^]^ Furthermore, MSNs, which harbor pro‐inflammatory factors such as free nucleic acids, can modulate the immune microenvironment and thus facilitate wound healing. Overall, the PFG/M MN system had a good biocompatibility and exhibited excellent antimicrobial and immunomodulatory properties, making it a promising strategy for healing infected wounds.

## 2 Results and Discussion

### 2.1 Preparation and Characterization of NPs

**Figure 1:**
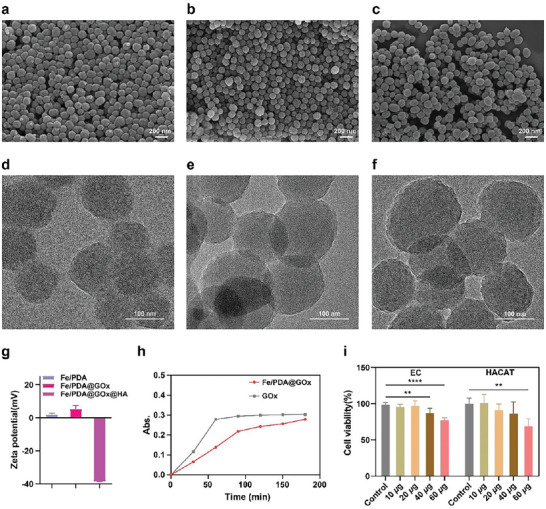
SEM images of a) Fe/PDA, b) Fe/PDA@GOx, and c) Fe/PDA@GOx@HA. TEM images of d) Fe/PDA, e) Fe/PDA@GOx, and f) Fe/PDA@GOx@HA. g) Zeta potential of Fe/PDA, Fe/PDA@GOx, and Fe/PDA@GOx@HA. h) Absorption intensity at *λ* = 405 nm for the analysis of H_2_O_2_ production. i) Biocompatibility evaluation of Fe/PDA@GOx@HA using CCK‐8 assay (*n* = 3). Error bars indicated means ± standard deviation (SD). Statistical significances were analyzed using one‐way ANOVA. ** *p* < 0.01, **** *p* < 0.0001.

Polydopamine‐loaded iron (Fe/PDA) was obtained as described previously.^[^ ^12^ ^]^ As shown in **Figure** **1**a,d, Fe/PDA had an average diameter of 110 nm and a high degree of uniformity. GOx was then transplanted onto the surface of Fe/PDA via acylation.^[^ ^12^ ^]^ To optimize the water stability and dispersibility of Fe/PDA@GOx, HA was coated on the surface by electrostatic adsorption. The resulting Fe/PDA@GOx and Fe/PDA@GOx@HA had average diameters of 150 nm (Figure 1b,e) and 170 nm (Figure 1c,f), respectively. Transmission electron microscopy (TEM) showed that the surface of Fe/PDA was coated with a thin shell (Figure 1e,f). The two shell layers were obviously shown on the surface of Fe/PDA by transmission pattern, which further proved that GOx and HA were successfully encapsulated on the surface of NPs (Figure S1, Supporting Information). As shown in Figure 1g, the negative potential of Fe/PDA@GOx@HA was higher than those of Fe/PDA and Fe/PDA@GOx, indicating that HA was successfully coated on the surface of the NP. Furthermore, the results of dynamic light scattering showed that Fe/PDA, Fe/PDA@GOx, and Fe/PDA@GOx@HA maintained good dispersity (Figure S2, Supporting Information).

In addition, the glucose‐oxidase‐like catalytic properties of Fe/PDA@GOx@HA were assessed. Since glucose oxidase converts glucose into gluconic acid and H_2_O_2_, the concentration of the produced H_2_O_2_ was quantified using TiSO_4_ with a characteristic absorption peak at 405 nm to confirm the catalytic properties (Figure S3, Supporting Information). The results showed that the amount of H_2_O_2_ gradually increased over time (Figure 1h), demonstrating the glucose oxidase‐like catalytic properties of Fe/PDA@GOx@HA. We confirmed whether the addition of HA affected the catalytic activity of GOx in Fe/PDA@GOx@HA. The results showed that HA protected the activity of GOx and slightly affected the enzyme activity (Figure S4, Supporting Information). The peroxidase (POD) activity of Fe/PDA@GOx@HA was examined, and the •OH produced via the Fenton reaction was examined using electron paramagnetic resonance with DMPO to capture •OH. A four‐line peak was observed in the Fe/PDA@GOx@HA‐DMPO system (Figure S5, Supporting Information), indicating the POD properties. The release process of Fe/PDA@GOx@HA under high GSH and acidic conditions, as well as the changes in the activity of each component after release were tested. TEM image showed the NP decomposition in a weak acid and high GSH environment for 48 h (Figure S6, Supporting Information). The results in Figure S7, Supporting Information showed that the GOx activity slightly decreased with the decomposition of Fe/PDA@GOx@HA. Furthermore, we assessed the biocompatibility of Fe/PDA@GOx@HA using the Cell Counting Kit‐8 (CCK‐8) assay. As shown in Figure 1i, the viabilities of human umbilical vein endothelial cell (EC) and human keratinocyte (HACAT) were more than 90% when the concentration of Fe/PDA@GOx@HA was not more than 20 µg mL^−1^, demonstrating excellent biocompatibility.

The MSNs were prepared using a templating method.^[^ ^14^ ^]^ After removing the surfactant, the MSNs were amine‐functionalized with 3‐aminopropyltriethoxysilane (APTES) to achieve a better bonding performance.^[^ ^15^ ^]^ The ninhydrin reaction resulted in a blue‐violet color after treatment, indicating the successful amino‐functionalization of MSNs (Figure S8a, Supporting Information). Scanning electron microscopy (SEM) demonstrated that the AP‐MSNs were uniformly dispersed NPs with a particle size of ≈150 nm (Figure S8b, Supporting Information), and TEM analysis confirmed the mesoporous channels of the AP‐MSNs (Figure S8c, Supporting Information). We tested the specific surface area and the average pore size of the mesopores of MSN by BET specific surface area test (Figure S9, Supporting Information). The average pore size of MSN was 3.22592 nm. Meanwhile, the surface of MSN was modified with amino groups, which would greatly enhance the adsorption capacity of MSN. We then tested the adsorption capacity of AP‐MSN for nucleic acids to reduce persistent inflammatory responses. As shown in Figure S10, Supporting Information, the adsorption of nucleic acids was linearly related to the amount of AP‐MSN, indicating that 100 µg AP‐MSNs could adsorb 1340 ng nucleic acids. Furthermore, we assessed the cytotoxicity of AP‐MSN using the CCK‐8 assay. The cell viabilities of HUVEC and HACAT were more than 90% when the concentration of AP‐MSN was not more than 400 µg mL^−1^, indicating negligible cytotoxicity of AP‐MSN (Figure S11, Supporting Information).

### 2.2 Photothermal Properties of Fe/PDA@GOx@HA

**Figure 2:**
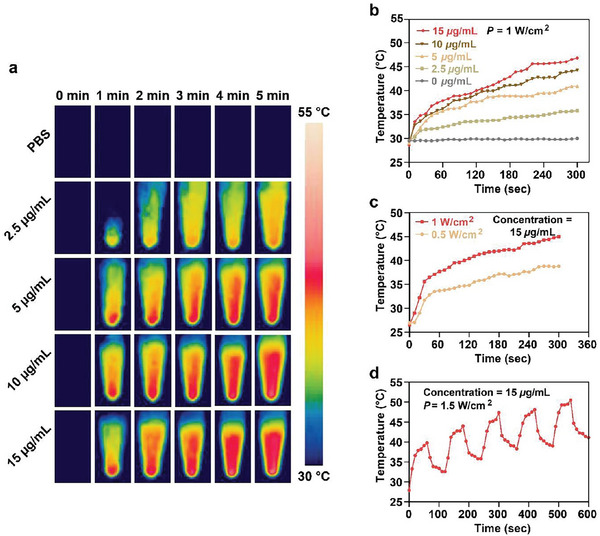
a) Real‐time infrared thermal images and b) photothermal curves of Fe/PDA@GOx@HA solutions at various concentrations. c) Photothermal curves of Fe/PDA@GOx@HA solutions under different laser power intensity. d) Photothermal curves of 15 µg mL^−1^ Fe/PDA@GOx@HA solution in five on/off cycles.

We assessed the photothermal properties of Fe/PDA@GOx@HA in vitro using various NP concentrations and laser intensities. Fe/PDA@GOx@HA (0, 2.5, 5, 10, and 15 µg mL^−1^ in PBS) were continuously irradiated with a 660 nm laser at 1 W cm^−2^ for 5 min. As shown in **Figure** **2**a,b, the photothermal efficiency was positively correlated with the NP concentration. The temperature of 15 µg mL^−1^ Fe/PDA@GOx@HA solution reached as high as 46.8 °C, while the temperature of the PBS was maintained at 30 °C. Figure 2c shows that the temperature of Fe/PDA@GOx@HA increased in a laser power intensity‐dependent manner. The temperature reached as high as 46.8 and 38.8 °C at the laser power of 1 W cm^−2^ and 0.5 W cm^−2^, respectively. The photothermal properties of Fe/PDA@GOx@HA remained stable during five on/off cycles of laser heating (Figure 2d), supporting its use as a photothermal agent in repeated PTT. These results demonstrate that temperature elevation can be controlled by modulating the Fe/PDA@GOx@HA concentration and laser power density. The heat generated by Fe/PDA@GOx@HA was sufficient for bacterial elimination. To test the photothermal capacities after decomposition, 15 µg mL^−1^ Fe/PDA@GOx@HA was dispersed into a PBS solution containing 10 mm GSH with a pH of 6.5 for 48 h. The solution was continuously irradiated with a 660 nm laser at 1 W cm^−2^ for 5 min. The results showed that the photothermal properties of Fe/PDA@GOx@HA slightly decreased with the decomposition (Figure S12, Supporting Information).

### 2.3 Preparation and Characterization of the PFG/M MN System

**Figure 3:**
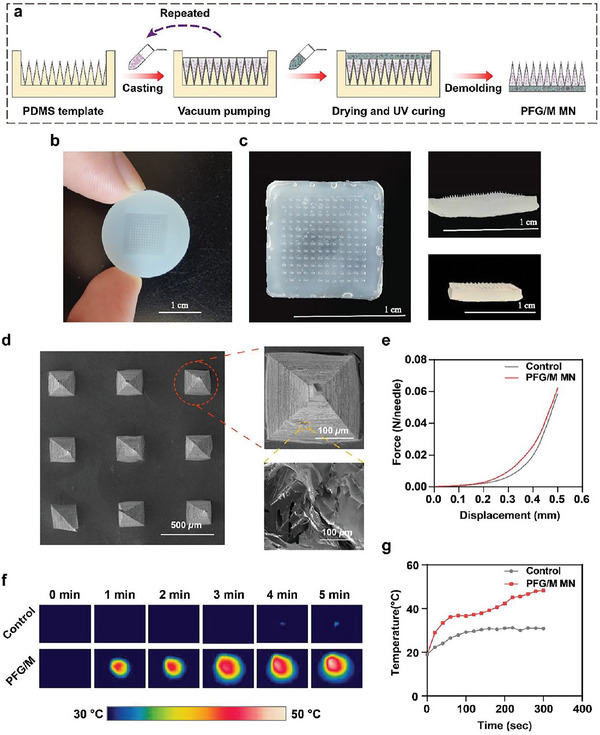
a) Schematic illustration of the preparation of the PFG/M MN patch. b) Optical image of the PDMS mold. c) Optical image of the PFG/M MN patch. d) SEM image of the PFG/M MN patch. e) Force‐displacement curves of the MNs. f) Infrared thermal images and g) photothermal curves of the MNs irradiated with a 660 nm laser at 1 W cm^−2^ for 5 min.

Biofilms serve as a major factor that limits the effects of antimicrobial biomaterials. MN can penetrate and disrupt the integrity of biofilms, resulting in diffusion of antibacterial agents.^[^ ^9^ ^]^ To ensure the efficient delivery of Fe/PDA@GOx@HAs and AP‐MSNs to the infected wound, the two NPs were loaded into a hyaluronate methacrylate (HAMA) MN system. A PFG/M MN system was prepared using a template‐based method (**Figure** **3**a).^[^ ^14^ ^]^ In detail, Fe/PDA@GOx@HAs and AP‐MSNs were thoroughly mixed with the HAMA solution. The HAMA solution containing Fe/PDA@GOx@HAs was cast into a negative polydimethylsiloxane (PDMS) mold (Figure 3b). The tips were filled with HAMA solution containing Fe/PDA@GOx@HAs using a vacuum pump. The excess HAMA solution was removed using a surgical blade. The above two steps were repeated twice to completely fill the needle tips with the HAMA solution. The AP‐MSN solution was then added to the mold to form the base of the MN patch. Finally, the PFG/M MN patch was obtained via UV solidification.

The PFG/M MN contained a 12 × 12 MN array (Figure 3c). The height, base axis, and center‐center width of the needle were 650, 280, and 750 µm, respectively. The SEM images show quadrangular pyramidal tips (Figure 3d). A compression experiment was performed to assess the mechanical strength of PFG/M MN. The needles were not fractured during the experiment and could withstand compressive forces (0.06 N per needle) (Figure 3e), which were higher than the minimum force required to penetrate the human skin.^[^ ^16^ ^]^ Then, we assessed the capability of piercing the PFG/M MN patches into the excised mouse skin. Hematoxylin and eosin (HE) staining of mouse showed insertion sites (Figure S13, Supporting Information), indicating the good penetration ability of the PFG/M MN. Furthermore, we determined the photothermal properties of PFG/M MN. As shown in Figure 3f,g, the temperature of the PFG/M MN irradiated with 660 nm laser at 1 W cm^−2^ for 5 min gradually increased to 48.2 °C, proving that the PFG/M MN possessed the photothermal ability with the potential to kill bacteria. The temperature of the control MN reached 30.9 °C and showed little photothermal effect under the same circumstances.

### 2.4 Biocompatibility Evaluation

**Figure 4:**
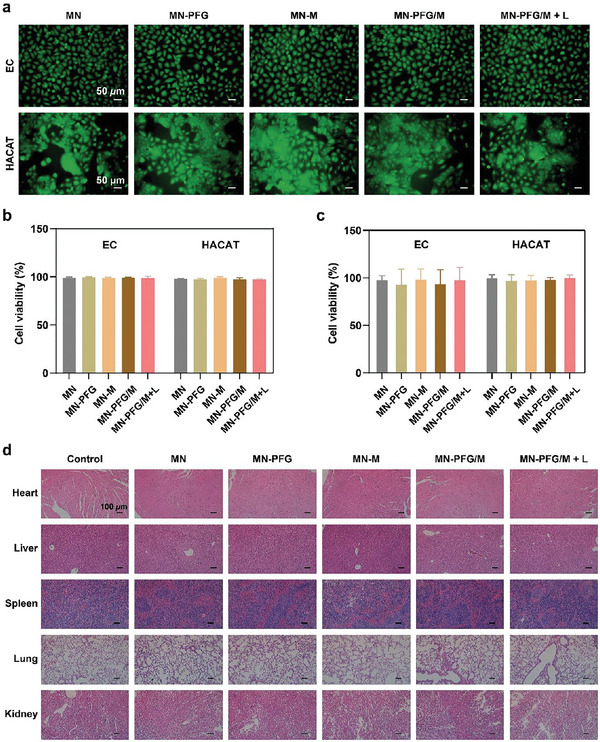
a) Fluorescence images and b) quantitative analysis of live/dead cells of EC and HACAT using Calcein‐AM/PI assay (*n* = 3). The cells were cocultured with the media extracts of MNs, treated with or without a 660 nm laser at 1 W cm^−2^ for 5 min, and then further incubated for 24 h. c) Quantitative analysis of CCK‐8 assay (*n* = 3). The cells were cocultured with the media extracts of MNs and treated with or without a 660 nm laser at 1 W cm^−2^ for 5 min, followed by further incubation for 24 h. d) H&E staining of the main organs (heart, liver, spleen, lung, and kidney) from mice with infected wound on day 14 after treatment. Error bars indicated means ± SD. Statistical significances were analyzed using one‐way ANOVA.

Biosafety is a basic requirement for biomaterial applications, and ECs and keratinocytes are the two main cell types involved in wound healing.^[^ ^17^ ^]^ Therefore, we further evaluated the biocompatibility of the PFG/M MN patch and validated the cytotoxicity of the MNs in HACAT and ECs in vitro using CCK‐8 and the calcein‐AM/propidium iodide (Calcein‐AM/PI) staining assays. As shown in **Figure** **4**a,b, the results of the calcein‐AM/PI assay suggested that the cell viabilities of EC and HACAT in all MN groups with or without laser irradiation remained above 95%, indicating good biocompatibility. This was further confirmed by the results of CCK‐8 assay (Figure 4c). Next, we estimated the hemocompatibility of MNs *ex vivo* using a hemolysis test. The supernatant of the positive control group remained red and transparent with a hemolysis ratio (HR) of ≈100%. The supernatants from the four MN groups were similar to those of the negative control group. The HRs were 0.50%, 0.56%, 0.56%, 1.63%, and 0.20% for the MN, MN‐PFG, MN‐M, MN‐PFG/M, and the negative control group, respectively (Figure S14, Supporting Information). An HR of < 5% has been reported to be permissible.^[^ ^18^ ^]^ Finally, we estimated the biocompatibility of the MNs in vivo. The main organs were collected from mice with infected wounds on day 14 after treatment, and an HE staining assay was performed. As shown in Figure 4d, no obvious inflammatory lesions or tissue damage were observed in the heart, liver, spleen, lung, or kidney of any of the groups, which further confirmed the excellent biosafety of the MN‐PFG/M system. Taken together, our results indicate that PFG/M MN possesses good biocompatibility.

### 2.5 In Vitro Antibacterial Performance and Immunomodulating Property

**Figure 5:**
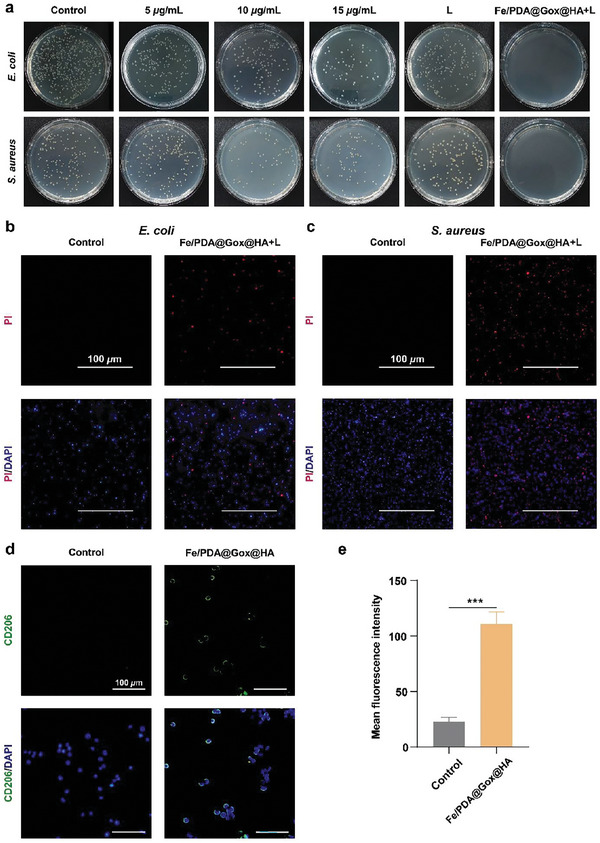
a) Colony formation by *E. coli* and *S. aureus*. Bacteria were cocultured with various concentrations of the Fe/PDA@GOx@HA, irradiated with or without a 660 nm laser at 1 W cm^−2^ for 5 min, and then incubated for another 24 h. b) Live/dead bacterial staining images of *E. coli* and c) *S. aureus* after incubation with 15 µg mL^−1^ Fe/PDA@GOx@HA and irradiation with a 660 nm laser at 1 W cm^−2^ for 5 min, followed by incubation for another 24 h. d) Fluorescence images and e) quantitative analysis of RAW 264.7 cells after treatment with LPS (control) or LPS/Fe/PDA@GOx@HA (*n* = 3). CD206: M2 macrophages. Error bars indicated means ± SD. Statistical significances were analyzed using student's *t*‐test. *** *p* < 0.001.

Bacterial infections are one of the main causes of non‐healing wounds. Therefore, antibacterial property is crucial in wound dressings. Next, we validated the antibacterial performance and immunomodulatory properties of these biomaterials in vitro. *Staphylococcus aureus* (*S. aureus*, gram‐positive) and *Escherichia coli* (*E. coli*, gram‐negative) were selected to assess the antibacterial capability of Fe/PDA@GOx@HA. As shown in **Figure** **5**a, the number of colonies gradually decreased as the concentration of Fe/PDA@GOx@HA increased compared to the control group, suggesting that Fe/PDA@GOx@HA killed bacteria in a dose‐dependent manner. Compared with the Fe/PDA, Fe/PDA@GOx@HA and L groups, after irradiation with a 660 nm laser at 1 W cm^−2^ for 5 min, 15 µg mL^−1^ Fe/PDA@GOx@HA possessed excellent antibacterial capability, and no colony was formed (Figure 5a, Figure S15, Supporting Information). This suggests that the CDT and PTT of Fe/PDA@GOx@HA exhibited a synergistic effect. Antibacterial capability was further confirmed using a live/dead bacterial staining assay. PI dye can only penetrate dead bacteria with destroyed structure and fluoresces red under excitation, while the 4′,6‐diamidino‐2‐phenylindole (DAPI) binds to the DNA of live and dead bacteria and fluoresces blue under excitation. As illustrated in Figure 5b,c, in the Fe/PDA@GOx@HA+L group, the percentage of red fluorescence was significantly higher than that in the control group, demonstrating that Fe/PDA@GOx@HA could kill *S. aureus* and *E. coli* under 660 nm laser irradiation. Bacterial biofilm is a structured bacterial community embedded in a self‐produced extracellular polymeric substance.^[^ ^19^ ^]^ Since biofilm formation is an important cause of persistent bacterial infection that significantly inhibits wound healing, we tested the antimicrobial capability of Fe/PDA@GOx@HA toward biofilms. As illustrated in Figure S16, Supporting Information, biofilm formation was significantly inhibited in the Fe/PDA@GOx@HA+L group. These results demonstrate that Fe/PDA@GOx@HA has excellent antimicrobial activity against both planktonic bacteria and biofilms.

Immune dysregulation also impairs wound healing, and the sustained presence of a pro‐inflammatory response is a characteristic of chronic wounds.^[^ ^20^ ^]^ Modulating the immune microenvironment is an effective treatment strategy for chronic wounds.^[^ ^7^ ^]^ In the wound bed, M1 macrophages produce pro‐inflammatory cytokines, whereas M2 macrophages have an anti‐inflammatory phenotype and secrete anti‐inflammatory mediators. PDA, which has excellent biocompatibility, can inhibit M1 polarization and promote M2 polarization.^[^ ^13^ ^]^ We next validated the immunomodulatory properties of Fe/PDA@GOx@HA. CD86 and CD206 are surface molecules expressed on M1 and M2 macrophages, respectively. CD206 expression significantly increased in the Fe/PDA@GOx@HA group, while CD86 expression significantly decreased in the Fe/PDA@GOx@HA group (Figure 5d,e, Figure S17, Supporting Information). Interleukin‐10 (IL‐10) was one of the most important anti‐inflammatory cytokines secreted by M2 macrophages. The pro‐inflammatory cytokines, interleukin‐6 (IL‐6) and tumor necrosis factor‐𝛼 (TNF‐𝛼), were mainly secreted by M1 macrophages. The results showed a significant increase of IL‐10 in the Fe/PDA@GOx@HA group than that in the control group (Figure S18, Supporting Information). In contrast, IL‐6 and TNF‐𝛼 were significantly inhibited in the Fe/PDA@GOx@HA group (Figure S18, Supporting Information). These results suggest that Fe/PDA@GOx@HA promotes M2 macrophage polarization and inhibits M1 macrophage polarization, thus favoring an anti‐inflammatory response. Furthermore, we investigated whether the microneedles had the function of regulating macrophages. As shown in Figure S19, Supporting Information, the MN‐PFG/M group showed the best efficacy in increasing the CD206 expression and inhibiting the CD86 expression. These results indicate that MN‐PFG/M favored an anti‐inflammatory response.

### 2.6 PFG/M MN Accelerates Infected Wound Healing through Sterilization In Vivo

**Figure 6:**
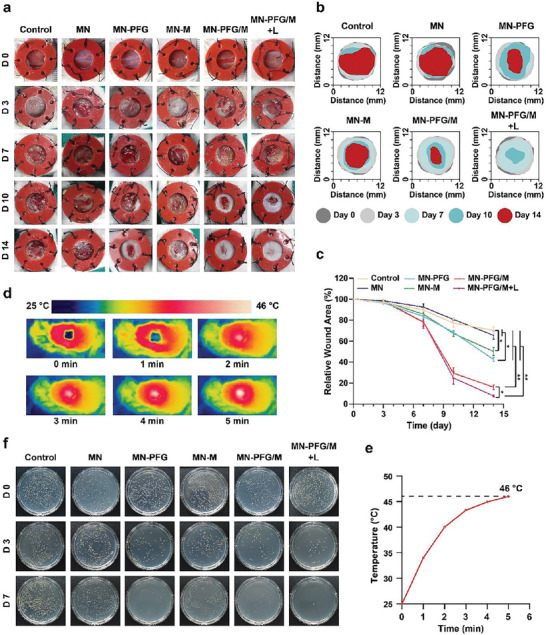
a) Representative images of the infected wounds and b) simulation of the process of wound healing. c) Quantitative analysis of the wound area (*n* = 6). d) Photothermal images and e) thermal curve of the wound treated with MN‐PFG/M plus NIR. f) Representative images of bacterial colony in wound fluid. Error bars indicated means ± SD. Statistical significances were analyzed using one‐way ANOVA. * *p* < 0.05, ** *p* < 0.01.

The wound healing efficacy of PFG/M MN was further validated using bacteria‐infected wound models in vivo. We selected the *S. aureus*‐infected wound model for further validation because *S. aureus* is a major cause of wound infections.^[^ ^21^ ^]^ The infected wound model was established and processed as illustrated in Figure S20, Supporting Information. The wound conditions were recorded on days 0, 3, 7, 10, and 14. As illustrated in **Figure** **6**a,b, the wound closure rates in the MN‐PFG/M and MN‐PFG/M+L groups were higher than those in the other groups. Quantitative analysis revealed that the relative wound areas decreased to 70.3%, 65.6%, 42.6%, 50.2%, 16.1%, and 7.8% in the control, MN, MN‐PFG, MN‐M, MN‐PFG/M, and MN‐PFG/M+L groups, respectively (Figure 6c). These results indicate that the MN‐PFG/M and MN‐PFG/M+L groups were more favorable for infected wound healing. Furthermore, because CDT and PTT exhibited a synergistic effect, the MN‐PFG/M+L group exhibited better healing properties than the other groups. As illustrated in Figure 6d,e, after being irradiated with a 660 nm laser at 0.5 W cm^−2^ for 5 min, the working temperature of MN‐PFG/M increased to 46 °C, which had a mild PTT for killing bacteria without damaging the wound tissue.^[^ ^17^ ^]^ To confirm the antibacterial effect of MN‐PFG/M in vivo, we collected the wound fluid or biofilms and counted the number of bacteria using a colony formation assay on days 0, 3, and 7. As shown in Figure 6f, the number of bacterial colonies in the MN‐PFG/M and MN‐PFG/M+L groups significantly decreased, and few bacterial colonies were observed in the MN‐PFG/M+L group on day 7 after treatment. These results suggest that MN‐PFG/M exerts synergistic effects via CDT and PTT to kill bacteria and promote wound healing.

**Figure 7:**
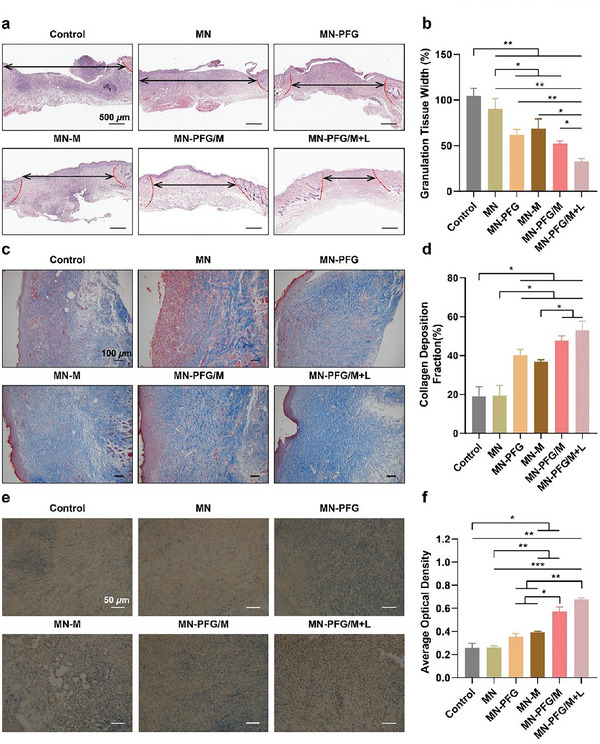
a) Representative images of HE staining of the wound bed. The double‐headed arrows indicate the granulation tissues gaps and wound edges. b) Qualification of granulation tissues gaps in (a) (*n* = 6). c) Masson staining and d) qualification of collagen deposition (*n* = 6). e) Immunohistochemistry staining and f) qualitative analysis of CD206 (*n* = 6). Error bars indicated means ± SD. Statistical significances were analyzed using one‐way ANOVA. * *p* < 0.05, ** *p* < 0.01, *** *p* < 0.001.

HE staining of the wound bed tissues was performed to assess wound healing after treatment. Tissue granulation and re‐epithelialization are indicators of the reconstruction phase of the wound healing process. The granulation tissue was located between the wound edges. A narrower granulation tissue space indicates faster wound closure and better wound healing.^[^ ^22^ ^]^ Epithelial cells migrate from the wound margins; thus, re‐epithelization occurs.^[^ ^23^ ^]^ As shown in **Figure** **7**a,b, the granulation tissue gap was significantly narrowed in the MN‐PFG/M+L group, and the re‐epithelialization process was completed in the MN‐M, MN‐PFG/M, and MN‐PFG/M+L groups. Furthermore, collagen deposition was denser and more organized in the MN‐PFG/M+L group (Figure 7c,d). Together, these results suggested that MN‐PFG/M+L treatment killed bacteria, stimulated wound closure, and promoted collagen deposition, thereby improving infected wound healing.

### 2.7 In Vivo Immunomodulating Property of PFG/M MN

Promoting the conversion of macrophages from a pro‐ to an anti‐inflammatory phenotype, thereby modulating the immune microenvironment, is an effective treatment strategy for chronic wounds.^[^ ^24^ ^]^ The inflammatory status, including macrophage polarization and inflammatory cytokines in the wound bed on day 14 after treatment, was examined using immunohistochemical staining. CD206 and CD86 are generally accepted markers for M2‐like and M1‐like macrophages, respectively. As shown in Figure 7d,f, the expression of CD206 was the lowest in the MN‐PFG/M+L group. CD86 expression was significantly inhibited in the MN‐PFG/M+L group (Figure S21, Supporting Information). These results suggested that MN‐PFG/M plus NIR treatment enhanced M2 macrophage polarization. Notably, M2 macrophage polarization in the MN‐PFG/M+L group was elevated compared to that in the MN‐PFG and MN‐M groups. Efficient antimicrobial strategies promote wound healing. However, pathogenic nucleic acid fragments released from degraded bacteria and necrotic cells elicit potent inflammatory responses, leading to prolonged inflammatory activation. The MN‐PFG/M patch exerted antibacterial effects and simultaneously adsorbed pro‐inflammatory factors such as free nucleic acids, thus efficiently inhibiting the pro‐inflammatory response.

Interleukin (IL)‐10 plays a significant role as an anti‐inflammatory mediator in wound healing. As shown in Figure S22, Supporting Information, IL‐10 expression was significantly higher in the MN‐PFG/M+L group, ensuring protection from excessive responses to infection. Besides, TNF‐*α* and IL‐6, two typical inflammatory cytokines, were diminished in the MN‐PFG/M+L group, which further confirmed the inhibited inflammatory response in the wound tissue (Figures S23 and S24, Supporting Information).

## 3 Conclusions

In summary, we designed and constructed an MN system (PFG/M MN) to promote infected wound healing. The MN system exhibits excellent antimicrobial and immunomodulatory properties. Our results demonstrate that PFG/M MN penetrated the bacterial biofilm, resulting in the diffusion of Fe/PDA@GOx@HA. Under 660‐nm laser irradiation, Fe/PDA@GOx@HA exhibited CDT and PTT, achieving a synergistic antimicrobial effect. PDA promoted M2 macrophage polarization. Furthermore, MSNs in the MN base adsorbed pro‐inflammatory factors such as free nucleic acids, inhibiting pro‐inflammatory response and thus improving wound healing. Overall, these features of the PFG/MN system indicate that it is a promising clinical candidate for healing infected wounds.

## Conflict of Interest

The authors declare no conflict of interest.

## Supporting information

Supporting Information

## Data Availability

The data that support the findings of this study are available from the corresponding author upon reasonable request.
