# Supplementary material for: Microneedle Patches with Antimicrobial and Immunomodulating Properties for Infected Wound Healing
Source: Adv Sci (Weinh). 2023 May 18;10(22):2300576. doi: 10.1002/advs.202300576 (PMC10401127; doi:10.1002/advs.202300576)
Supplement: Supplementary file 1 — Supporting Information [file ADVS-10-2300576-s001.pdf]

ADVANCED SCIENCE
Open Access

Supporting Information

for *Adv. Sci.*, DOI 10.1002/advs.202300576

Microneedle Patches with Antimicrobial and Immunomodulating Properties for Infected Wound Healing

Shengbo Li, Xuemei Wang, Zhiyao Yan, Tian Wang, Zhenbing Chen, Heng Song and Yongbin Zheng**

Supporting Information

Microneedle Patches with Antimicrobial and Immunomodulating Properties for Infected Wound Healing

Shengbo Li[#], *Xuemei Wang*[#], *Zhiyao Yan*, *Tian Wang*, *Zhenbing Chen*, *Heng Song*[*] *and Yongbin Zheng*[*]

S.B.Li, X.M.Wang, T.Wang, Prof. H.Song, Prof. Y.B.Zheng

Department of Gastrointestinal Surgery, Renmin Hospital of Wuhan University; College of Chemistry & Molecular Science, Institute of Molecular Medicine, Wuhan University, Wuhan 430060, China

E-mail: hengsong@whu.edu.cn, yongbinzheng@whu.edu.cn

Z.Y.Yan

Medical College, Hubei University of Arts and Science, Xiangyang 441000, China

Prof.Z.B.Chen

Department of Hand Surgery, Union Hospital, Tongji Medical College, Huazhong University of Science and Technology, Wuhan 430022, China

Materials

Hyaluronic acid, glucose oxidase (GOx), 5,5'-Dithiobis (2-nitrobenzoic acid) (DTNB), rhodamine B, and Cholesterol and Titanium sulfate (TiSO4) were received from Meryer in China. Hyaluronic acid methacrylic acid (HAMA, 150 kDa) was purchased from Engineering For Life company (Suzhou, China). Ammonia, tetraethyl orthosilicate (TEOS), and dopamine were obtained from Sinopharm Chemical Reagent. 1-(3-Dimethylaminopropyl)-3-ethylcarbodiimide Hydrochloride (EDCI) and ferric oxide (Fe_2O_3) were obtained from Aladdin (Shanghai, China). Dulbecco's modified Eagle's medium (DMEM) was obtained from Gibco (USA). Calcein-AM/PI cell viability/cytotoxicity assay kit and Cell Counting Kit 8 (CCK-8) were obtained from Beyotime (China). Phosphate buffer saline (PBS) was obtained from Boster (Wuhan, China). Immunohistochemical antibodies against IL-10, IL-6, TNF-α, CD206, CD86 were received from Abcam (MA, USA). Fetal bovine serum (FBS) was obtained from HYCEZMBIO in China.

Preparation and characterization of Fe coated polydopamine nanospheres (Fe/PDA), Fe/PDA@GOx, and Fe/PDA@GOx@HA

Fe/PDA was first fabricated according to our previously reported research.[1] Then, 10 mg Fe/PDA was evenly dispersed into 10 mL deionized (DI) water by ultrasound and 5 mg EDCI was added. After being magnetic stirred, 5 mg GOx was added. After reacting at 4 °C for 24 h, then the mixture was washed 3 times with water. The Fe/PDA@GOx obtained by centrifugation (10,000 rpm, 10 min and 4 °C) was washed with DI water to remove the unreacted proteins. To prepare Fe/PDA@GOx@HA, 1 mg

Fe/PDA@GOx was fully dispersed into 5 mL DI water by ultrasound, to which 15 mg HA was added. Stirred magnetically, the mixture reacted at 4 °C for 24 h. The Fe/PDA@GOx@HA was obtained and washed with DI water for several times to remove unreacted HA.

The above three nanoparticles were characterized the morphology and size using a transmission electron microscopy (TEM, JEM-2100 Plus, JEOL, Japan) and a scanning electron microscope (SEM; Zeiss Merlin Compact). The nanoparticle size

and surface charge were analyzed using dynamic light scattering (DLS).

Enzyme-like catalytic activity of Fe/PDA@GOx@HA

GOx catalyzed glucose to produce gluconic acid and H_2O_2. The concentration of H_2O_2 was quantified by the reaction of $TiSO_4$ (24%) with a characteristic absorption peak at 405 nm. We first prepared 24% TiSO4 in 50 mL DI water, then 8.33 mL H_2SO_4 was added. 0.2 mL Fe/PDA@GOx@HA (500 ppm) or GOx (500 ppm) solution was added into the glucose solution (100 mM), followed by 2.1 mL PBS was added. The above solution (100 μL) was mixed with 200 μL $TiSO_4$. Then, the absorbance at 405 nm was examined at 30 minutes intervals. In order to test whether the addition of HA affected the catalytic activity of GOx in Fe/PDA@GOx@HA, we dispersed Fe/PDA@GOx@HA (500 ppm) and Fe/PDA@GOx (500 ppm) into PBS at pH=7.4 and detected the absorption spectra. For POD activity, the •OH produced by Fenton reaction was examined using electron paramagnetic resonance (EPR). DMPO was used to capture the •OH.

Preparation and characterization of mesoporous silica nanoparticles (MSNs) and amine-modified MSNs (AP-MSNs)

0.2 g cetyltrimethylammonium bromide (CTAB) was dissolved in a mixture of deionized water (100 mL) and 2 M NaOH (0.8 mL). Subsequently, the mixture was vigorously stirred at 80 °C for 4 h. Then, 1.0 mL TEOS was added dropwise to the CTAB dispersion. After being magnetic stirred for 4 h, the white precipitate was collected by centrifugation (12,000 rpm, 15 min). The precipitate was rinsed twice with ethanol and vacuum-dried overnight at 45 °C. The obtained MSNs were dissolved in 100 mL ethanol hydrochloride solution (10 mL hydrochloric acid plus 90 mL ethanol) and then refluxed at 78°C for 12 h. After centrifugation, the collected MSNs were cleaned alternately with ethanol and DI water.

1 g MSNs were dispersed into 100 mL anhydrous toluene for activation for 2 h. 0.25 mL of APTES was then added. The reaction was refluxed at 110 °C for 20 h then cooled to 35 °C. The pellet was obtained by centrifugation at 12000 rpm for 20 min, and then redispersed into pure toluene and centrifuged again. The AP-MSNs were collected and dried overnight at 100 °C in vacuum to remove residual solvent.

The AP-MSNs were characterized the morphology and size using a TEM and a SEM. The amino-functionalization of MSN was confirmed using the ninhydrin reaction. In detail, a small amount of silica was added to 5 ml of buffer containing 5% ninhydrin. The mixture was heated in an oven at 110 °C for 15 min. The color change of the reaction was recorded. The specific surface area and the average pore size of the mesopores of AP-MSN was measured using BET specific surface area test.

Different amounts of AP-MSN (0, 25, 50, 75, 100 μg) were dispersed into 400 μl water, respectively. Then 2000 ng DNA were added and mixed at 4 °C for 24 h. After centrifugation, the supernatant was taken to detect the concentration of DNA. Then, the adsorption curve was plotted.

Fabrication and characterization of microneedles

Fe/PDA@GOx@HAs (15 μg/mL) and AP-MSNs (400 μg/mL) were thoroughly blended with HAMA solution (0.1 g/mL) containing 2-hydroxy-2-methylpropiophenone (HMPP, 1% v/w), respectively. Then, the HAMA solution containing Fe/PDA@GOx@HAs were casted into the negative polydimethylsiloxane (PDMS) mold. The tips were filled with the HAMA solution containing Fe/PDA@GOx@HAs by a vacuum pump. Then, the excess HAMA solution was removed using a surgical blade. The above two steps were repeated twice to fully fill the needle tips. The AP-MSN solution was filled into the mold to form the base of the MN patch. Finally, the PFG/M MN patch was solidified by UV (405 nm) irradiation for 40 seconds and then demolded.

The MNs were characterized the morphology using a SEM (FEI Quanta25). For mechanical properties, a single column material testing machine was used. Briefly, the flat-headed probe continuously pressed the needles of MN in the vertical direction for a 0.5 mm displacement, and recorded the compression force.

To evaluate the skin penetration *ex vivo*, the PFG/M MN was pressed into the mice dorsal skin for several min. Then, the MN was removed and the skin was fixed in 4% paraformaldehyde for 24 h. The fixed skin tissue was embedded in paraffin wax and sliced for hematoxylin and eosin (HE) staining.

***In vitro* photothermal property of Fe/PDA@GOx@HA and MN**

To examine the photothermal effect, the PFG/M MN and Fe/PDA@GOx@HA (2.5, 5, 10, and 15 μg mL^{-1} in PBS) in a 1.5 mL Eppendorf tube were continuously irradiated with a 660 nm laser (Blueprint, Beijing, China) for 5 min at different power (0.5 W/cm^2 and 1 W/cm^2). To test the photothermal capacities after decomposition, 15 μg mL^{-1} Fe/PDA@GOx@HA was dispersed into a PBS solution containing 10 mM GSH with a pH of 6.5 for 48 h. The solution was continuously irradiated with a 660 nm laser at 1 W cm^{-2} for 5 min. The pure MN and PBS were served as the respective control groups. The photothermal stability of Fe/PDA@GOx@HA was assessed after laser irradiation. 15 μg mL^{-1} of Fe/PDA@GOx@HA in PBS was irradiated with a 660 nm laser for five on/off cycles. A FORTRIC225 thermal camera was used to record the photothermal data.

Cell culture

The human umbilical vein endothelial cells HUVECs, human keratinocytes (HACATs) and RAW 246.7 were cultured in DMEM (high glucose) containing 10% FBS under humidified atmosphere (5% CO_2, 37°C).

***In vitro* and *ex vivo* biocompatibility evaluation**

For in vitro cytotoxicity assay, HUVECs and HACAT cells were seeded into 96-well plates and cultured for 24 h. Then, different concentrations of Fe/PDA@GOx@HA or AP-MSN or the media extracts of the MNs were added to coculture for a certain time. The cytotoxicity was examined using the CCK-8 assay and the Calcein-AM/PI cell viability/cytotoxicity assay according to the manufacturer's instructions. For *ex vivo* biocompatibility evaluation, hemocompatibility assay was performed according to the previous reference.[2]

***In vitro* antimicrobial activity**

Colony formation assay were performed according to our previously reported research to assess the *in vitro* antimicrobial activity of the biomaterials.[3] PBS containing different concentrations of Fe/PDA@GOx@HA (0, 5, 10, and 15 μg mL^{-1}) was used to incubate *Escherichia coli* (*E. coli*) (Gram-negative, 10^6 CFU/mL) or *Staphylococcus aureus* (*S. aureus*, 10^6 CFU/mL) (Gram-positive) at 37 °C for 24 h. To examine the photothermal antimicrobial activity, 15 μg mL^{-1} Fe/PDA@GOx@HA

was added into the bacterial suspension and then irradiated with a 660 nm laser at 1 W cm^{-2} for 5 min. After incubated for 24 h, the bacteria were diluted with PBS. The suspension (100 μL) was plated on a LB agar, followed by incubation at 37 °C for 24 h. The colony formation was photographed.

For the live / dead bacterial staining assay, certain concentration of bacteria was stained using Propidium iodide (PI) dye and 4',6-diamidino-2-phenylindole (DAPI) at 37°C for 60 min. Then, 10 μL stained bacteria were dropped on a slide and taken photos using a fluorescence microscopy.

For biofilm formation experiment, 5×10^6 CFU/mL *E. coli* or *S. aureus* was cocultured with 0 or 15 μg mL^{-1} Fe/PDA@GOx@HA. After being irradiated with a 660 nm laser at 1 W cm^{-2} for 5 min, the bacterial suspension was incubated in a 96-well plate (200 μL per well) for 48 at 37 °C. The incubated wells were washed lightly with PBS. After fixed with 4% paraformaldehyde, the bacterial biofilm was stained with 0.5% crystal violet for 10 min, then washed with PBS for three times. The stained bacterial biofilm was treated with pure alcohol, then the absorbance at 590 nm was quantified.

***In vitro* immunomodulating ability**

RAW 264.7 macrophages on 12-well plates were treated with 0 or 15 μg mL^{-1} Fe/PDA@GOx@HA and 200 ng mL^{-1} lipopolysaccharide (LPS) for 24 h. RAW 264.7 cells were stained with anti-CD86 or anti-CD206 antibodies, then visualized by a fluorescence microscopy. The levels of interleukin-6 (IL-6), tumor necrosis factor-α (TNF-α), and interleukin-10 (IL-10) in the cell culture supernatants were measured using ELISA kits. To evaluate the effect of microneedle systems on the polarization of macrophages, Raw 264.7 macrophages were treated with 200 ng mL^{-1} LPS for 24 h. cocultured with nothing, MN, PFG MN, M MN, PFG/M MN, respectively.

Mouse model of infected wounds

The animal experiments were approved by the Animal Care Committee of Tongji Medical College. BALB/c (male, 20 – 30 g) mice were used as the model of infected wound. The 1 cm full-thickness round skin wound was made on the backs of BALB/c

mice. Then, 20 μL S. aureus suspension (1×10^8 CFU/mL) was dropped into the wound. The mice were randomly divided into six groups (n = 6): control group, MN group, MN-PFG group, MN-M group, MN-PFG/M group, MN-PFG/M+L group, which were treated with nothing, MN, PFG MN, M MN, PFG/M MN, and PFG/M MN plus NIR (660 nm laser at 0.5 W cm^{-2} for 5 min), respectively. The photothermal images in the MN-PFG/M+L group was recorded using A FORTRIC225 thermal camera. The treatments were repeated every 2 days. The wounds were captured on day 0, 3, 7, 10, and 14. The wound area was assessed using Image J software. The relative wound area was calculated referring to the previous report.[2]

In vivo biosafety evaluation

After treatment, the mice were sacrificed and the primary organs were obtained. The organs of mice were fixed and then stained with HE to observe the pathological changes.

In vivo antimicrobial effect of MN

We obtained the mixture of MN and scab from the infected wounds on day 0, 3, and 7. The mixture was diluted with PBS and incubated on LB agar for 24 h. The colony formation was captured.

Histological analysis

We obtained the wound bed tissues of the infected wound on day 14. HE and Masson staining were performed. Then, immunohistochemistry analysis was performed to assess the expression of CD206, CD86, IL-10, IL-6, and TNF-α, which were then quantified using Image J.

Statistical analysis

The data were presented with mean ± standard deviation (SD) based on at least three tests. The statistical analysis was performed using GraphPad Prism 8 (GraphPad, USA). Column statistics were performed to evaluate the normal distribution. Comparisons were performed using student’s t-test between two groups or using one-way ANOVA analysis among multiple groups. The significant difference defined as * ($p < 0.05$), ** ($p < 0.01$), *** ($p < 0.001$), and **** ($p < 0.0001$).

Figure S1 – S24

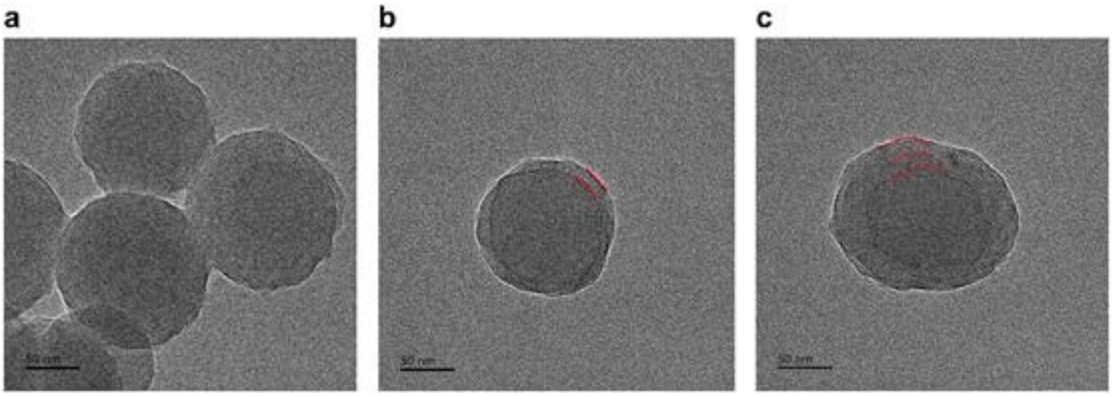

Figure S1. TEM images of (a) Fe/PDA, (b) Fe/PDA@GOx, and (c) Fe/PDA@GOx@HA.

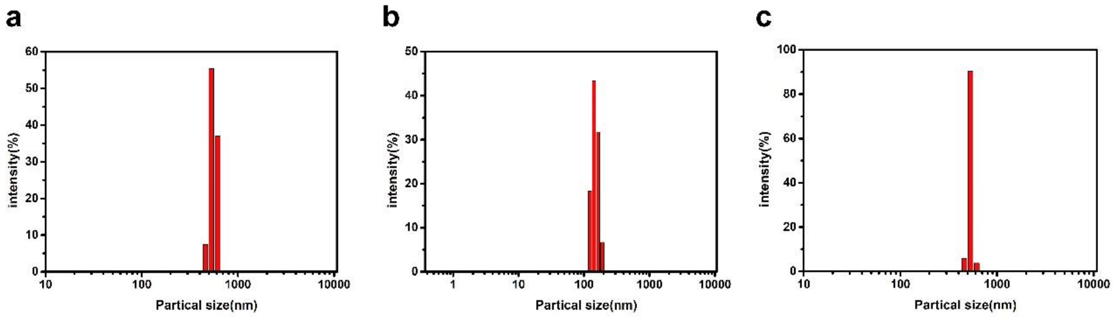

Figure S2. The size distribution of (a) Fe/PDA, (b) Fe/PDA@GOx, and (c) Fe/PDA@GOx@HA.

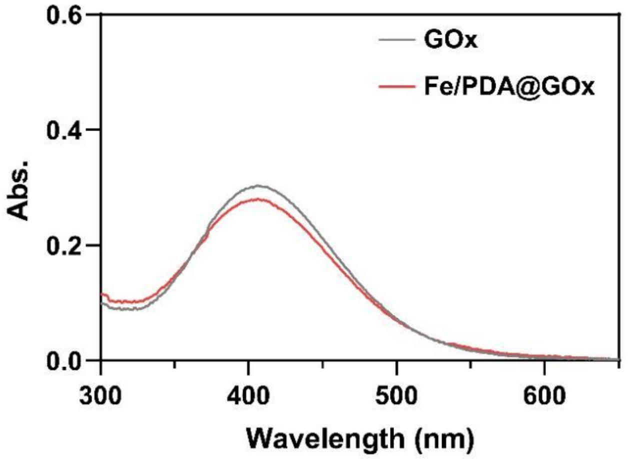

Figure S3. UV−vis absorption spectra of GOx activity.

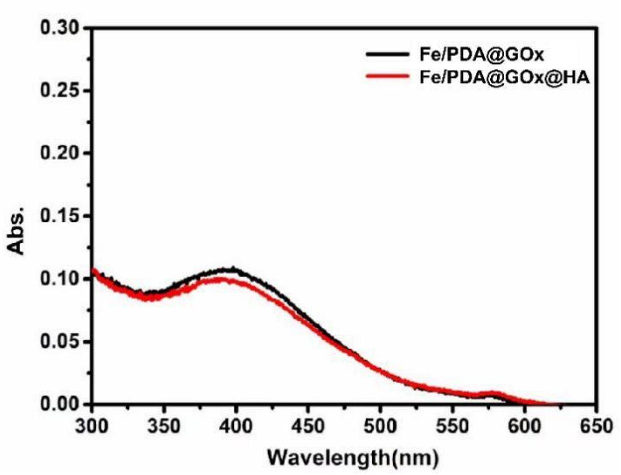

Figure S4. UV−vis absorption spectra of GOx activity of Fe/PDA@GOx and Fe/PDA@GOx@HA.

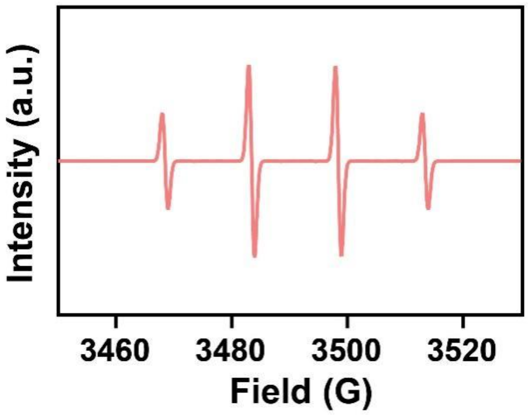

Figure S5. The EPR spectra of •OH generated in the H_2O_2 / Fe/PDA@GOx@HA / DMPO solution.

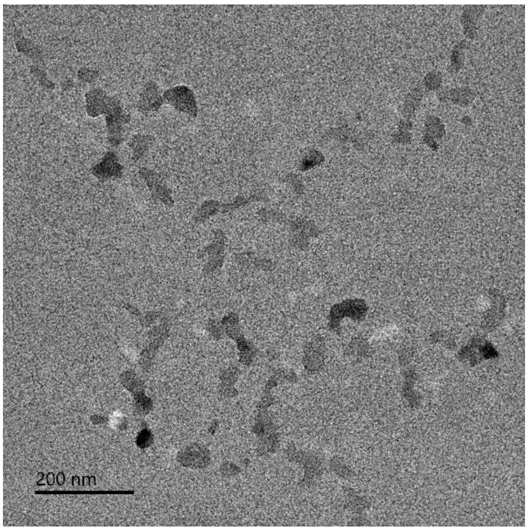

Figure S6. TEM image of Fe/PDA@GOx@HA under 10mM GSH conditions at 37 ℃ for 48 h.

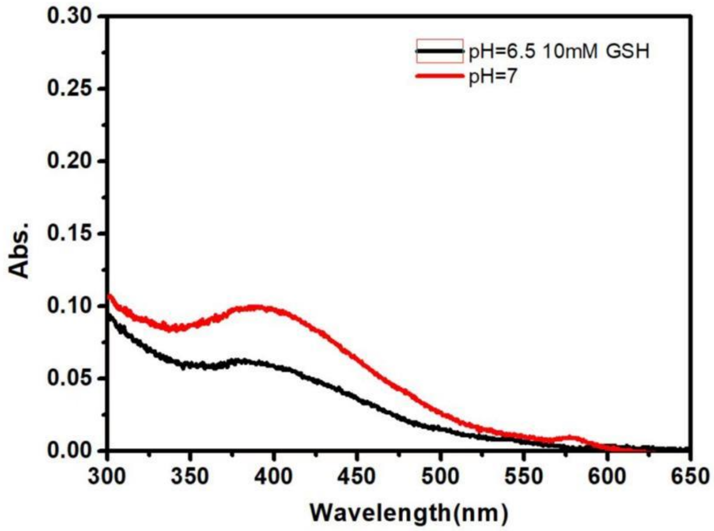

Figure S7. UV−vis absorption spectra of GOx activity of Fe/PDA@GOx@HA before (pH = 7) and after decomposition (pH = 6.5, 10 mM GSH).

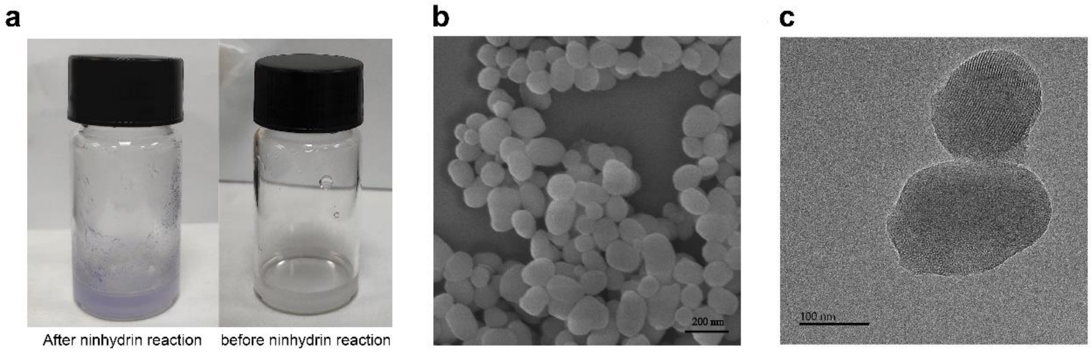

Figure S8. (a) Images of ninhydrin reaction of the amino-functionalized MSNs. (b) The SEM image of AP-MSNs. (c) The TEM image of AP-MSNs.

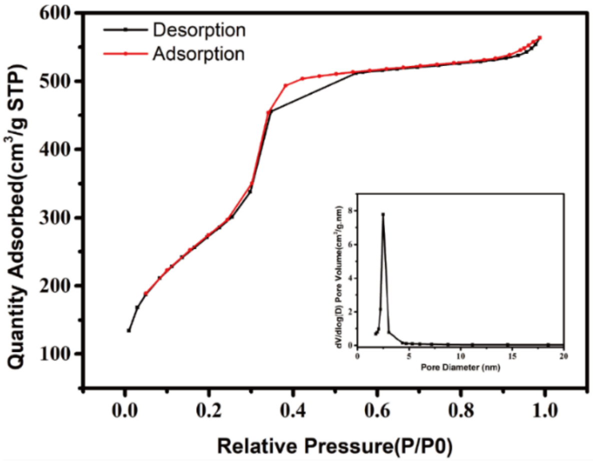

Figure S9. Nitrogen sorption isotherms and pore size distribution curves(inset) of AP-MSN.

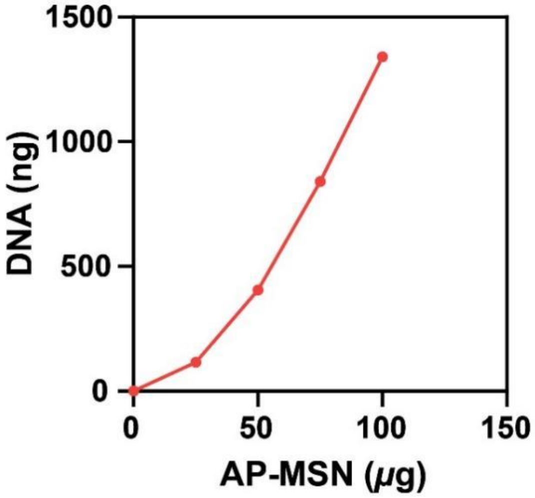

Figure S10. The nucleic acid adsorption curve for AP-MSN.

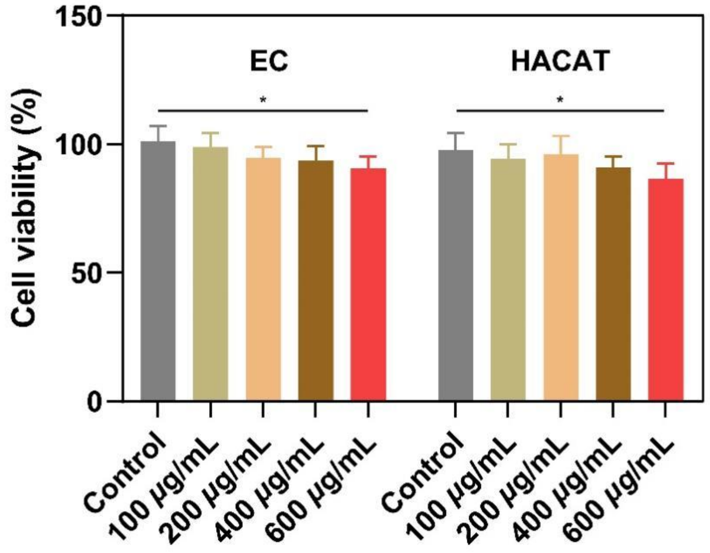

Figure S11. Biocompatibility evaluation of AP-MSN using CCK8 assay (n = 3). Error bars indicated means ± SD. Statistical significances were analyzed using one-way ANOVA. * $p < 0.05$.

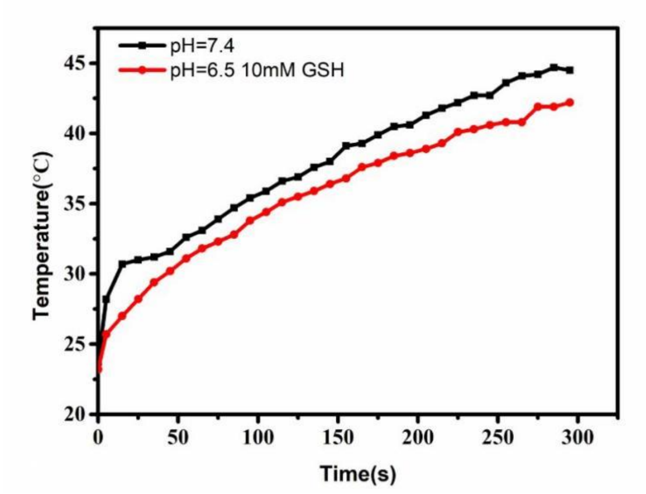

Figure S12. Photothermal curves of Fe/PDA@GOx@HA solutions before (pH = 7) and after decomposition (pH = 6.5, 10 mM GSH).

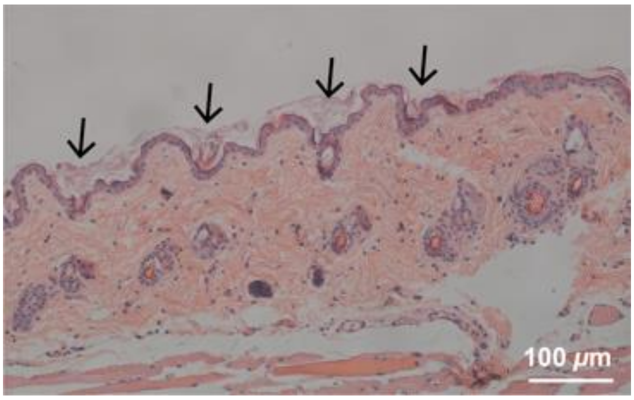

Figure S13. The image of H&E staining of mouse skin after applying PFG/M MN *ex vivo*.

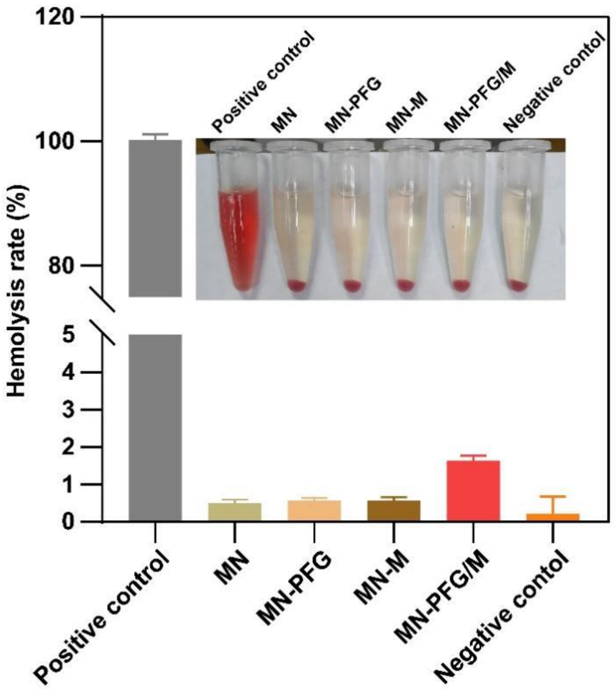

Figure S14. The hemolysis test of the MN patches *ex vivo* (n = 3). Error bars indicated means ± SD.

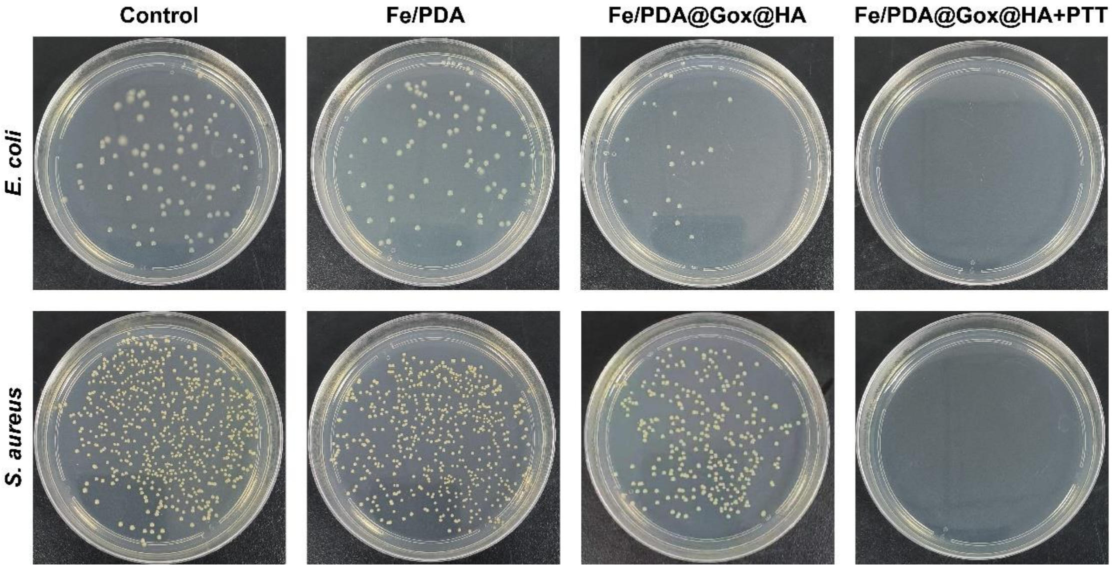

Figure S15. Colony formation by *E. coli* and *S. aureus*.

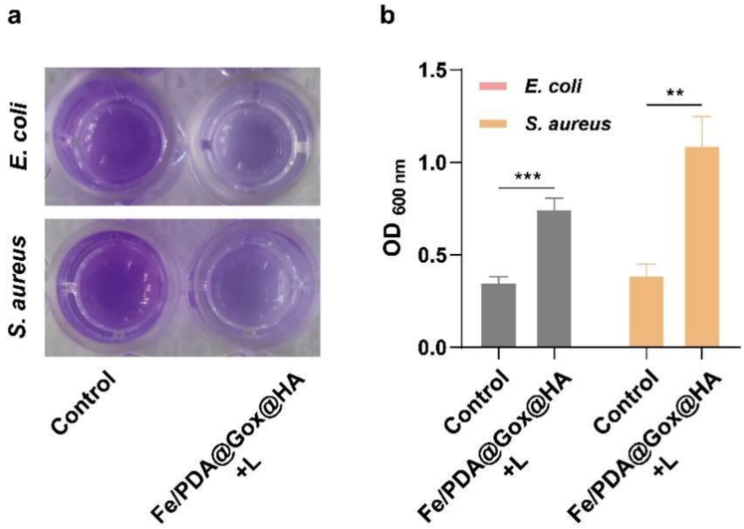

Figure S16. (a) Images and (b) quantitative analysis of biofilm formation after different treatments (n = 3). Error bars indicated means ± SD. Statistical significances were analyzed using student's t-test. ** $p < 0.01$, *** $p < 0.001$.

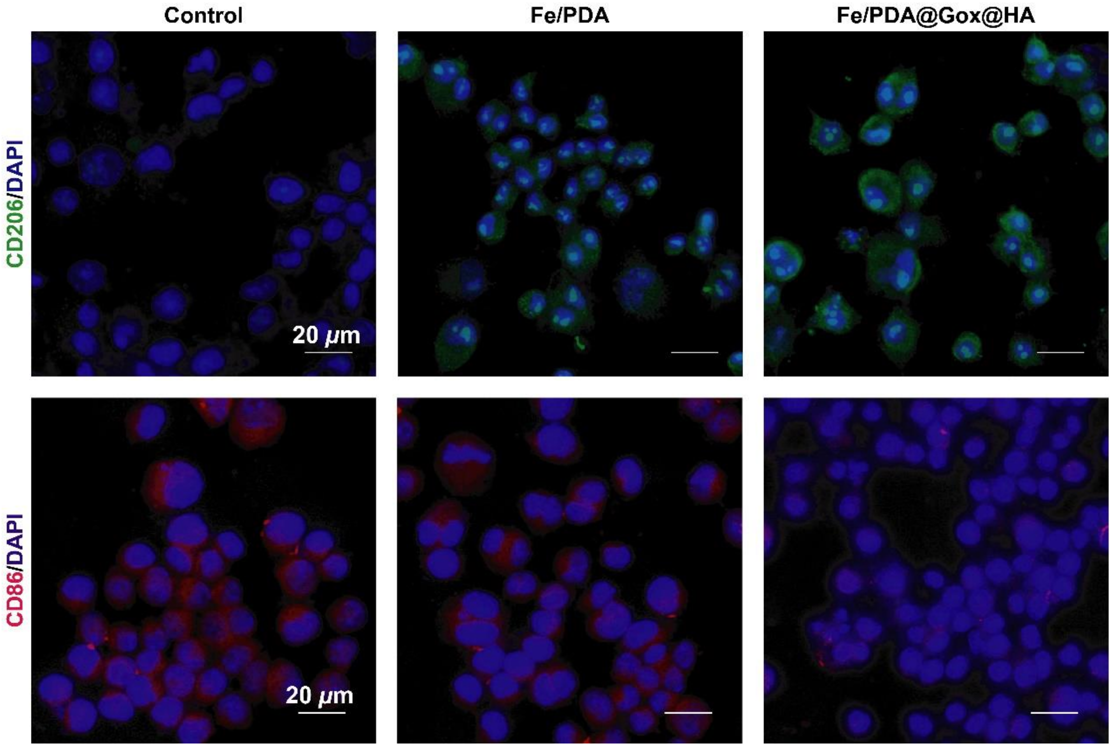

Figure S17. Fluorescence images of RAW 264.7 cells after being treated with the system of LPS (control) or LPS / Fe/PDA@GOx@HA. CD86: M1 macrophages.

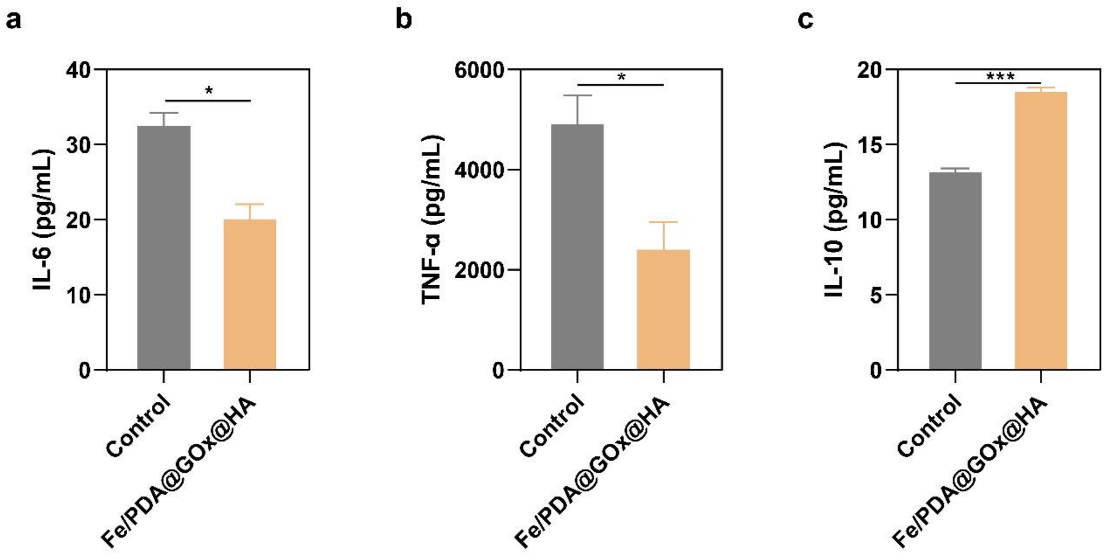

Figure S18. ELISA assay of (a) IL-6, (b) TNF-α, and (c) IL-10 in RAW 264.7 cell culture supernatants after being treated with the system of LPS (control) or LPS / Fe/PDA@GOx@HA (n = 3). Error bars indicated means ± SD. Statistical significances were analyzed using student's t-test. * p < 0.05, *** p < 0.001.

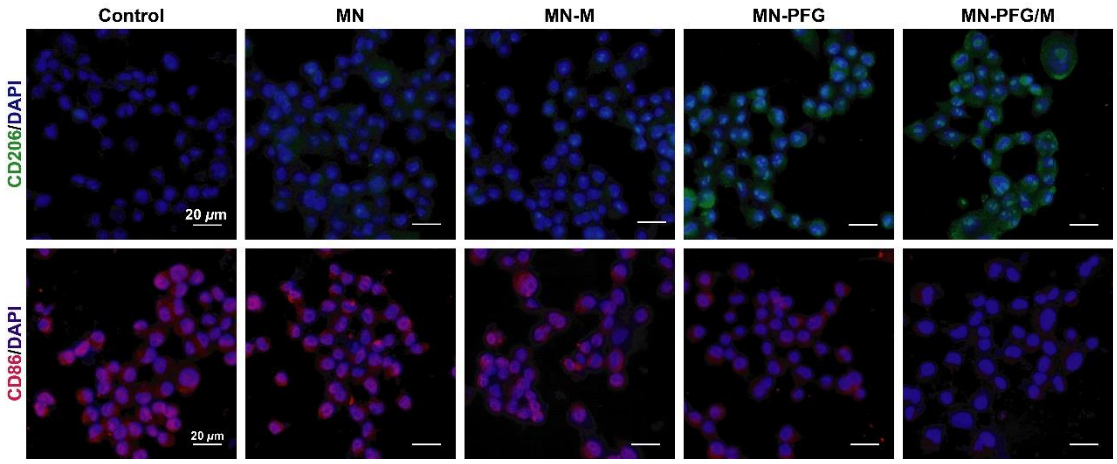

Figure S19. Fluorescence images of RAW 264.7 cells after being treated with the system of LPS (control) or LPS plus MNs. CD206: M2 macrophages, CD86: M1 macrophages.

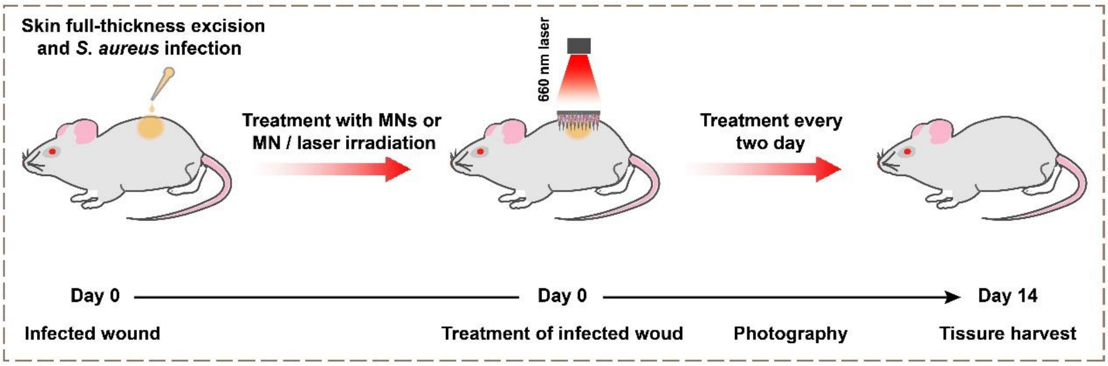

Figure S20. Experimental procedures showing the induction and treatment of the mouse infected wound.

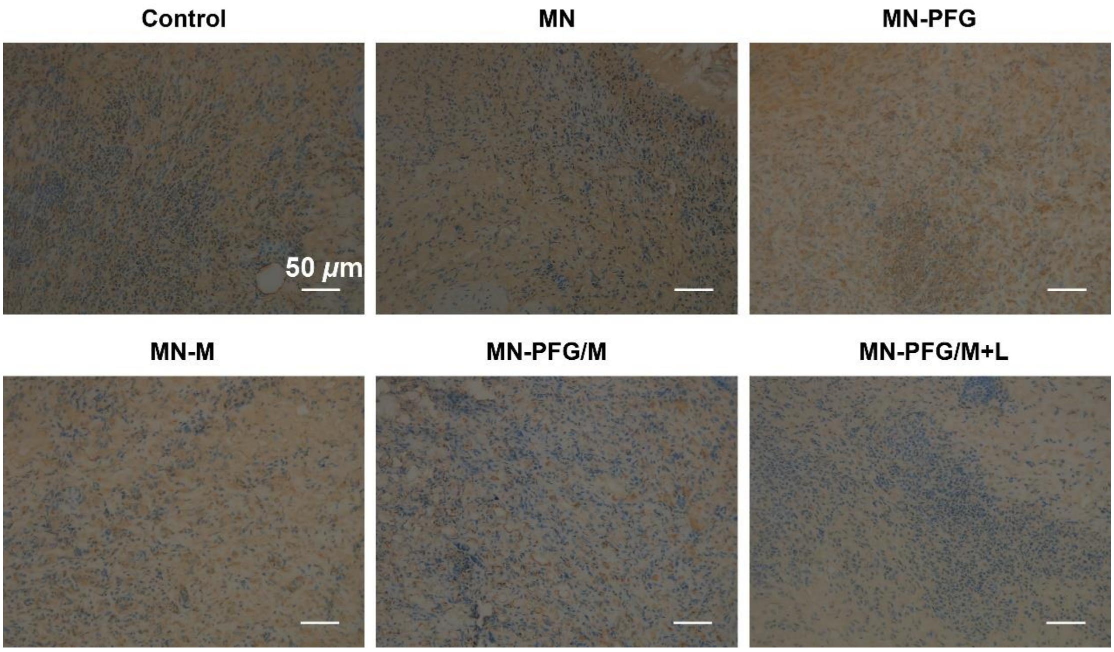

Figure S21. Immunohistochemistry staining of CD86.

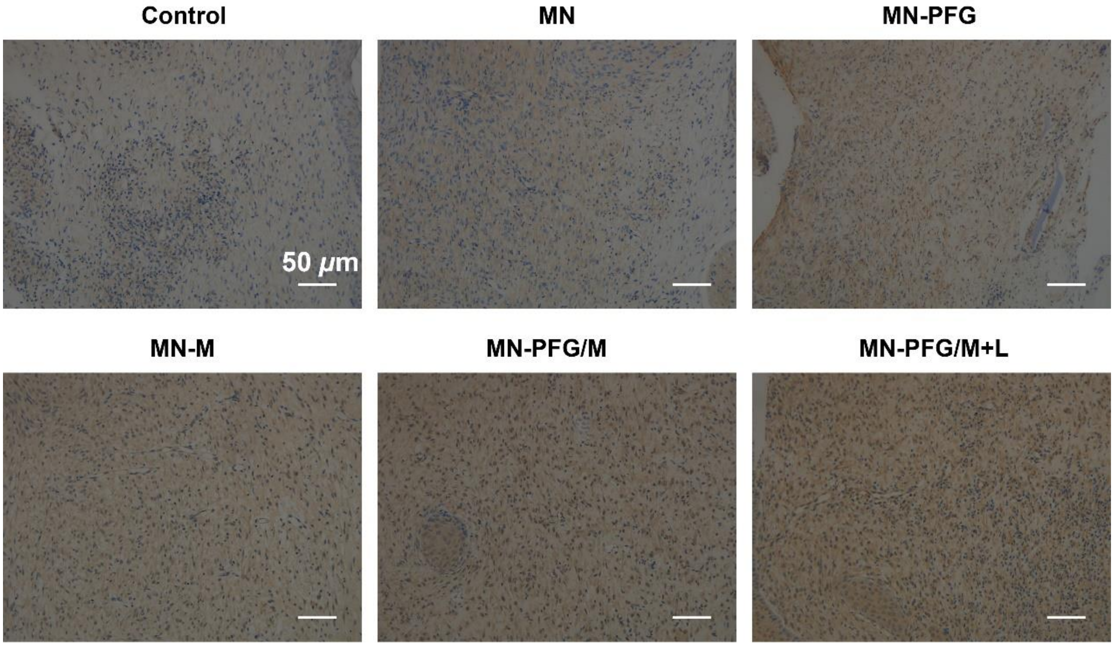

Figure S22. Immunohistochemistry staining of IL-10.

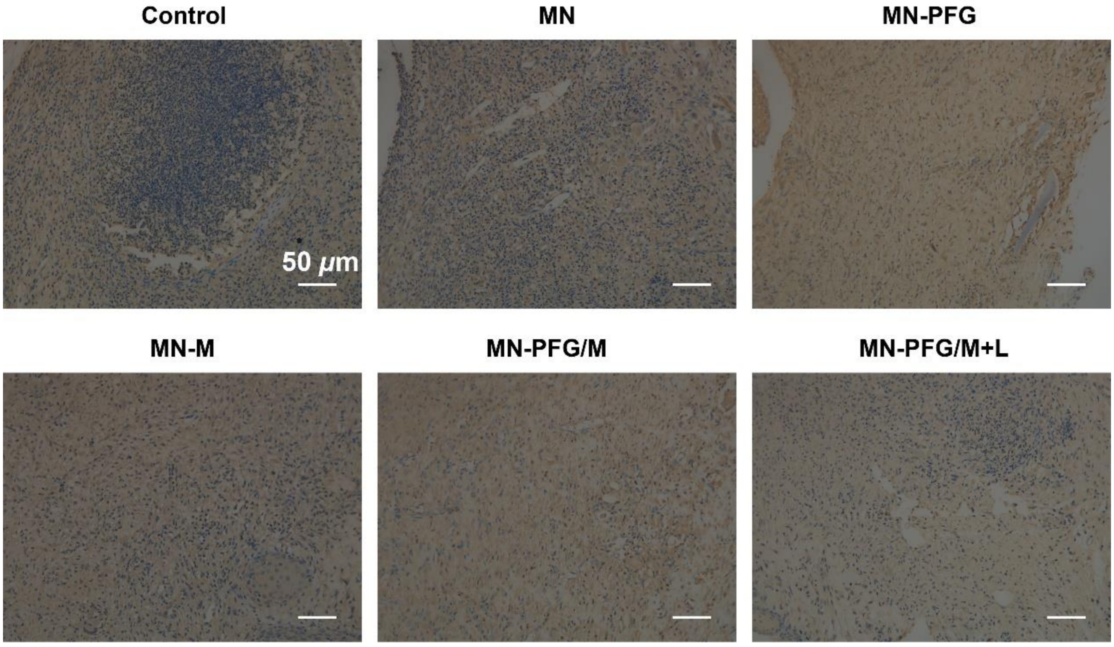

Figure S23. Immunohistochemistry staining of TNF-α.

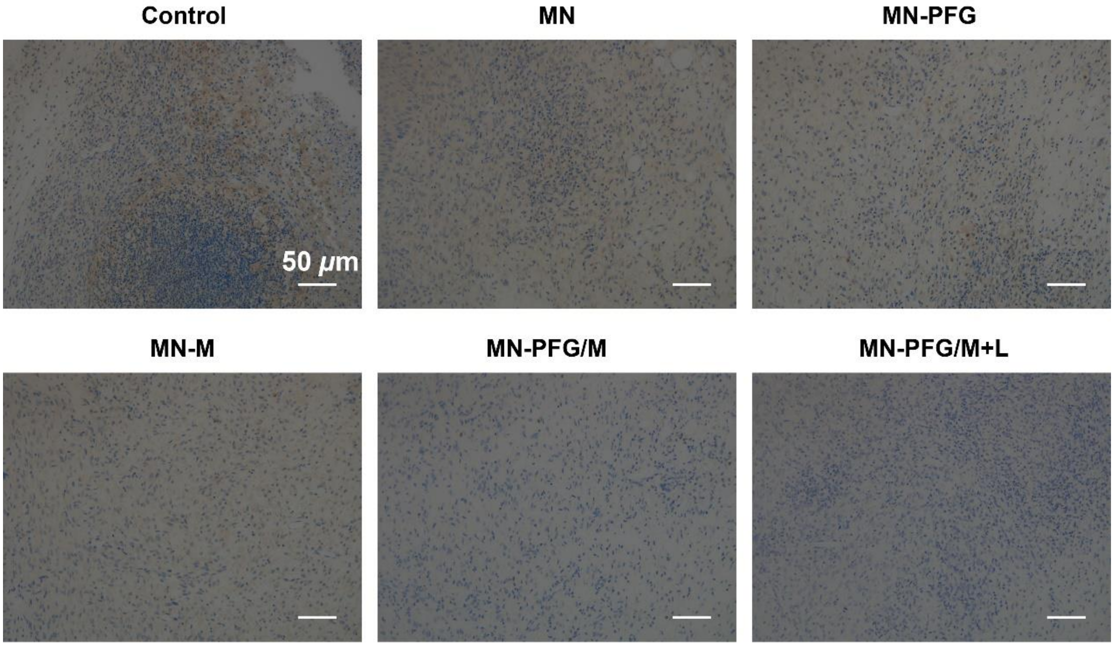

Figure S24. Immunohistochemistry staining of IL-6.

References:

[1] X. Wang, S. Li, S. Wang, S. Zheng, Z. Chen, H. Song, *Adv Sci (Weinh)* **2022**, e2202453.

[2] J. Chen, Y. Liu, G. Cheng, J. Guo, S. Du, J. Qiu, C. Wang, C. Li, X. Yang, T. Chen, Z. Chen, *Small* **2022**, 18, e2201300.

[3] S. Li, X. Wang, J. Chen, J. Guo, M. Yuan, G. Wan, C. Yan, W. Li, H. G. Machens, Y. Rinkevich, X. Yang, H. Song, Z. Chen, *Int J Biol Macromol* **2022**, 202, 657.
